# Using grass inflorescence as source material for biomonitoring through environmental DNA metabarcoding

**DOI:** 10.1007/s11033-024-09885-9

**Published:** 2024-09-16

**Authors:** Willem G. Coetzer

**Affiliations:** 1 Department of Zoology and Entomology, University of Fort Hare, Private Bag X1314, Alice, 5700 Eastern Cape South Africa; 2 Department of Genetics, University of the Free State, Bloemfontein, Free State South Africa

**Keywords:** High-throughput sequencing, Metabarcoding, Biodiversity assessment, Plant surface eDNA

## Abstract

**Background:**

Over the last decade, increasing attention has been directed to using different substrates as sources of environmental DNA (eDNA) in ecological research. Reports on the use of environmental DNA located on the surface of plant leaves and flowers have highlighted the utility of this DNA source in studies including, but not limited to, biodiversity, invasive species, and pollination ecology. The current study assesses grass inflorescence as a source of eDNA for detecting invertebrate taxa.

**Methods and results:**

Inflorescences from four common grass species in a central South African grassland were collected for high-throughput sequencing analysis. Universal COI primers were utilised to detect Metazoan diversity. The sequencing results allowed for the detection of three Arthropoda orders, with most OTUs assigned to fungal taxa (Ascomycota and Basidiomycota). Some biases were detected while observing the relative read abundance (RRA) results.

**Discussion:**

The observed biases could be explained by the accidental inclusion of invertebrate specimens during sample collection and DNA extraction. Primer biases towards the amplified taxa could be another reason for the observed RRA results. This study provided insight into the invertebrate community associated with the four sampled grass species. It should be noted that with the lack of negative field controls, it is impossible to rule out the influence of airborne eDNA on the observed diversity associated with each grass species. The lack of the inclusion of PCR and extraction blanks in the sequencing step, as well as the inclusion of negative field controls, including other areas for refinement were highlighted, and suggestions were provided to improve the outcomes of future studies.

**Supplementary Information:**

The online version contains supplementary material available at 10.1007/s11033-024-09885-9.

## Introduction

The use of DNA metabarcoding in studies related to molecular ecology and biodiversity has grown substantially over the years [1]. This method is a useful tool for biodiversity monitoring of small and elusive organisms, and has been successfully used in pollination studies [2], identifying vertebrate and invertebrate diets through metabarcoding of faecal matter or gut content [3], invasion biology [4] and monitoring of animal and plant communities [5, 6]. Environmental DNA (eDNA) can be detected from various substrates, ranging from marine and freshwater ecosystems using sediment and water samples, soil and plant material [7].

Recent advances in molecular techniques are allowing the use of an expanding list of possible substrates available for eDNA metabarcoding-driven biomonitoring studies. Reports showed how vertebrate biodiversity can be assessed using bulk insect samples [8]. The use of faecal samples to determine general biodiversity in an ecosystem has also been demonstrated [8, 9], with faecal samples from generalist omnivore species identified as a valuable source of eDNA, capable of detecting plant, vertebrate and invertebrate taxa found in the study area [9]. Several studies have recently reported on the utility of airborne eDNA to assess terrestrial biodiversity [10]; and references therein]. These various techniques and available substrates open a wide range of research possibilities for biodiversity studies in difficult-to-navigate terrain or studies focusing on elusive taxa. These methods can also provide an alternative for quick initial surveys to allow for the planning of future in-depth studies, therefore saving valuable time.

Collecting eDNA from plant surfaces is one novel method that has received attention from molecular ecologists over the last few years. A review by Banerjee et al. [11] highlights several examples of studies focussing on plant-animal interactions using eDNA methods. The study by Thomsen and Sigsgaard [12], for example, demonstrated the breadth of biodiversity information obtainable from plant surface eDNA. Flowers from different plant species were sampled across the study region, and the authors were able to detect a large amount of invertebrate diversity, with plant species-specific patterns identified [12]. Such information can assist in studying insect-plant pollination and food networks (plant–parasitoid or plant-herbivore) and detect interactions not easily observed by the eye [13]. Monge et al. [14] reported on the isolation of avian DNA from partially consumed fruits, and showed how the extracted eDNA is suitable for avian frugivore species identification and population genetics. The use of plant leaf material to detect a wide array of vertebrate and invertebrate taxa was reported by van der Heyde et al. [8] while testing the utility of different substrates for use in eDNA metabarcoding studies. eDNA metabarcoding is also an excellent tool for detecting invasive plant pests [15] and providing an effective means of invasive pest control management.

Globally, grasslands comprise 40% of terrestrial land area [16]. Grassland biomes provide crucial ecosystem services to the human communities living in and around these areas (e.g. grazing of livestock, collection of native plants and hunting) [17], and play critical ecosystem functions (e.g. carbon sequestration, water regulation and climate regulation) [18]. Large portions of South Africa are covered by the Grassland biome (16.5%), with the major plant taxon being grasses (Poaceae) [19, 20]. Grasslands are highly biodiverse habitats, which supports the ecosystem services and functions observed for these biomes [21]. Despite being such a crucial ecosystem, South African grasslands are not receiving the level of conservation attention which is needed [20, 21], and the multifunctional roles grasslands play in the ecosystem is quite understudied [18].

Here, I report on a pilot study to detect invertebrate visitations to grasses in the central Free State Province of South Africa using eDNA metabarcoding. Grasses are mainly wind-pollinated, but some cases of insect pollinators have been reported [22]. Many invertebrates also feed on grasses, and their predator species will also be present on grass surfaces, potentially leaving some eDNA behind. An unintentional outcome of the study included a large amount of fungal sequence data, providing additional information on the fungal community composition of the selected grass species. This pilot study will provide insight into possible areas that need optimisation for future larger-scale studies.

## Materials and methods

### Sample collection

**Fig. 1:**
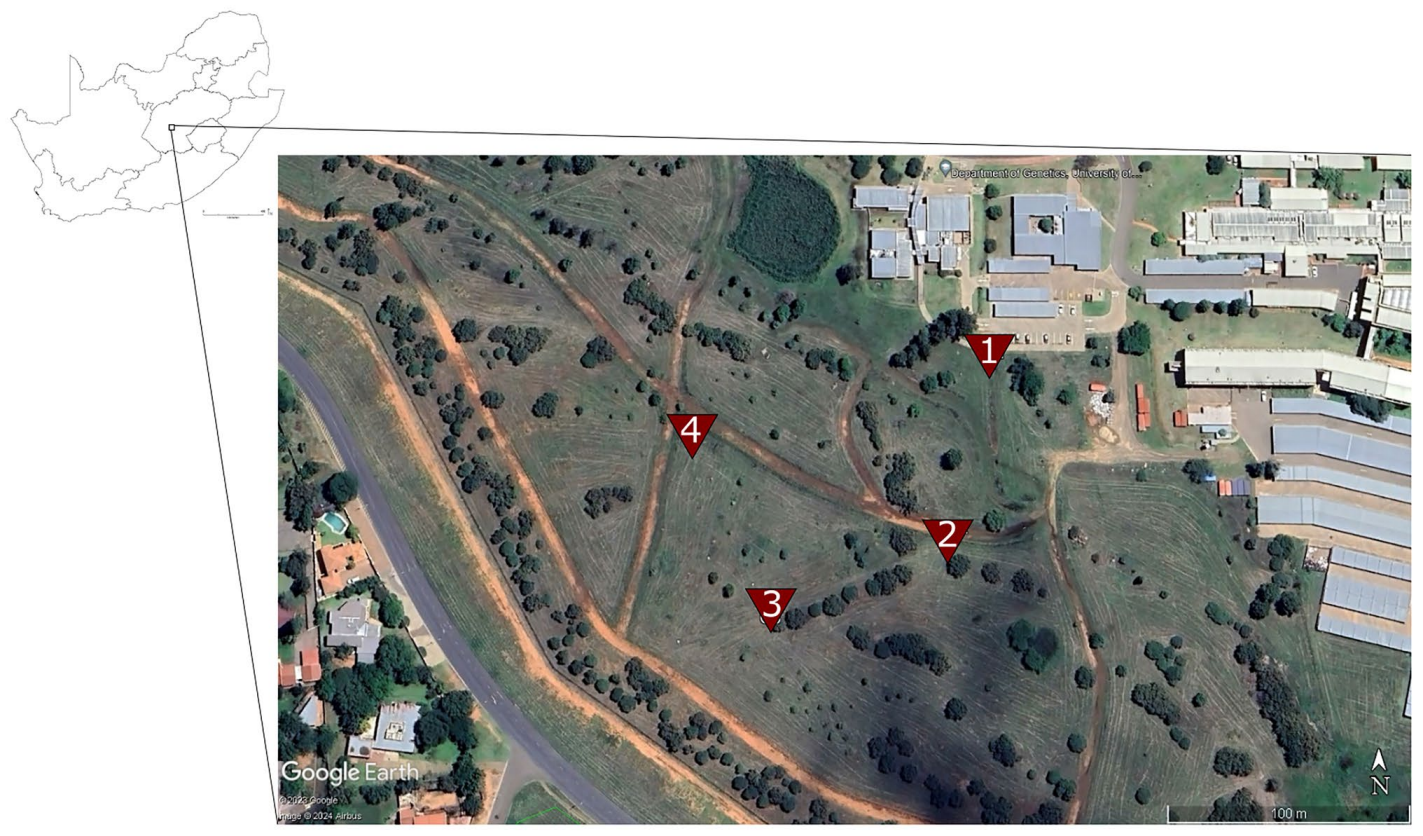
The location of the four samples sites within an urban grassland habitat at the University of the Free State Bloemfontein campus grounds. Native trees (*Searsia lancea*, *Acacia* (*Vachellia*) *karroo*, *Olea europaea* subsp. c*uspidate*) planted as windbreaks are interspersed within the grassland. The map was sourced from Google [24]. (Color figure online)

Samples were collected from an urban grassland habitat on the University of the Free State (UFS) campus, Bloemfontein (Free State Province, South Africa). This region falls in the Dry Highveld Grassland Bioregion of the Grassland biome [19]. Several species of native trees (*Searsia lancea*, *Acacia* (*Vachellia*) *karroo*, *Olea europaea* subsp. c*uspidate*), planted as windbreaks, are interspersed within this urban grassland. Four grass species common to the area were selected. Inflorescence were collected from two species with false panicles (*Cymbopogon caesius* and *Themeda triandra*) and two species with open panicles (*Panicum coloratum* and *Sporobolus fimbriatus*) [23] within a 13 000 m^2^ area on the UFS, Bloemfontein campus (Fig. 1; Table 1). Four plants were sampled per grass species from four sites within the sampling block (n = 16 samples), four samples per grass species across all sites. Sample sites were, on average, 103.5 m apart (75–140 m). Sampled grasses at each site were between 8–12 m apart. Three to four spikelets (~ 4 cm long) were collected from each plant for the false panicle species. Three to four inflorescence branches were collected from each plant of open panicle species, as the individual spikelets are small in these species. New sterile gloves were used between sampling events, and the forceps and scissors were sterilized with 1.25% hypochlorite and 96% ethanol before each sample collection. Samples from each plant were placed in separate sterile 15ml plastic screw cap tubes, taking care not to include any invertebrates. The 16 samples were named FCC.1- FCC.4 (*Cymbopogon caesius*), FTT.1- FTT.4 (*Themeda triandra*), FPC.1- FPC.4 (*Panicum coloratum*) and FSF.1- FSF.4 (*Sporobolus fimbriatus*). The tubes were placed in a cooler bag with an ice pack while in the field and then moved to – 20 °C storage until DNA extraction. No field blanks were included.

**Table 1:** The GPS coordinates for the sample sites where all grass inflorescence samples were collected. Inflorescence from all grass species targeted for this study were sampled per site. The study sites are situated in an urban grassland setting at the University of the Free State, Bloemfontein, South Africa

Sample sites	Lat	Long	Sample ID	Grass species:
Site 1	− 29.111826	26.18056	FCP.1	*Cymbopogon caesius*
			FPC.1	*Panicum coloratum*
			FSF.1	*Sporobolus fimbriatus*
			FTT.1	*Themeda triandra*
Site 2	− 29.112546	26.18039	FCP.2	*Cymbopogon caesius*
			FPC.2	*Panicum coloratum*
			FSF.2	*Sporobolus fimbriatus*
			FTT.2	*Themeda triandra*
Site 3	− 29.112795	26.17966	FCP.3	*Cymbopogon caesius*
			FPC.3	*Panicum coloratum*
			FSF.3	*Sporobolus fimbriatus*
			FTT.3	*Themeda triandra*
Site 4	− 29.112155	26.17927	FCP.4	*Cymbopogon caesius*
			FPC.4	*Panicum coloratum*
			FSF.4	*Sporobolus fimbriatus*
			FTT.4	*Themeda triandra*

### DNA extraction

Surface genomic DNA was extracted from each sample using the Purelink Genomic DNA Kit (Life Technologies ™, Carlsbad, CA), following the manufacturer’s protocol for Buccal swab DNA extraction, with modifications. Subsamples of two spikelets for each false panicle sample, and two pieces of inflorescence branches, ~ 1.5 cm in length, for each open panicle sample were used for DNA extraction. The subsamples were placed in a 1.5 ml microcentrifuge tube containing 180 µl PureLink™ Genomic Digestion Buffer and 20 µl Proteinase K, ensuring the sample was completely immersed in the buffer mixture. The samples were incubated at 55 °C for 1 h, and the manufacturer’s protocol was thereafter followed. The DNA extractions were performed in two batches, one with all false panicle grass species and one with all open panicle grass species. Each sample was manually inspected to ensure no observable invertebrates were present before DNA extraction. A negative control extract was performed with each batch to assess any DNA contamination during the laboratory procedures. All work surfaces and equipment were sterilized using a 1.25% hypochlorite solution before and after each DNA extract. The DNA extracts, including the negative control, were assessed via gel electrophoresis on a 2% agarose gel. The negative control showed no signs of DNA on the gel and was not included in the remainder of the workflow. Nucleotide purity and quantity measurements were taken via spectrophotometry using a NanoDrop® Spectrophotometer ND-1000 (Thermo Fisher Scientific, DE, USA). The DNA extracts were stored at − 20 °C.

### DNA amplification and high-throughput sequencing

All DNA extracts were diluted to ~ 50 ng/µl and sent to MR DNA laboratories (Shallowater, TX, USA) for high throughput sequencing (HTS). A 313 bp fragment of the COI barcode gene was targeted using the primers Sauron [5’-GGDRCWGGWTGAACWGTWTAYCCNCC-3’; 25] and jgHCO2198 [5’- TAIACYTCIGGRTGICCRAARAAYCA-3’; [26] following in-house protocols at MR DNA laboratories. Sample specific barcodes were attached to the forward primers (Supplementary Table S1). Two PCR replicates were performed per sample, with no-template controls included in each PCR reaction. The PCR amplification success was evaluated by gel electrophoresis on a 2% agarose gel. The PCR amplicons were pooled and purified using calibrated Ampure XP beads (Beckman Coulter, Brea CA, USA), followed by an Illumina DNA library preparation protocol based on Rennstam Rubbmark et al. [25]. The PCR blanks were not included in the sequencing process. All HTS were performed on an Illumina MiSeq system following the manufacturer’s guidelines at MR DNA laboratories. All raw sequences are stored under BioProject PRJNA935099 (Assession numbers SAMN33297822-SAMN33297837) on the NCBI GenBank database.

### Sequence analysis

Raw data processing was performed using QIIME2 [27]. In summary, the data was first demultiplexed, and primers were then removed using the cutadapt plug-in [28]. The dada2 plug-in, with command *denoise-paired* and using the pseudo pooling method, was used to trim the reads only to include high-quality sequences (quality score > 30), whereafter the reads were denoised, merged and chimeras removed [29]. Amplicon sequence variants (ASVs) with an overall sequence abundance of less than ten were removed from the feature table and sequence files using the *feature-table filter-features* and *filter-seqs* commands. The dataset was further curated using the R package LULU v.0.1.0 prior to taxonomic identification to reduce the number of erroneous ASVs [30]. To simplify species identification, all curated ASVs were clustered to operational taxonomic units (OTUs) through de novo clustering using the *vsearch cluster-features-de-novo* command in QIIME2.

### Taxonomic assignment

The *classify-consensus-blast* feature classifier was used in QIIME2 for taxonomic assignment, using the MIDORI2 [31] Unique COI database (vGB260; released 15 April 2024). The percentage identity was set at 80%, and query coverage at 85%. A similar approach for the percentage identity setting was recently implemented by Mugnai et al. [32]. The authors suggested that a high identity threshold would be more suited to studies where the target taxa are well represented in the reference databases. The identity threshold of 0.8 selected in the current study is based on the assumption that South African representative sequences, such as those from Arthropods, are not well represented in online databases [33]. Curated Arthropoda and Fungi datasets were then created using the filtering options in QIIME2.

### Statistical analysis

Rarefaction curves were plotted for each dataset (Arthropoda and Fungi) per sample using the vegan package [34] in R [35]. Each dataset was rarefied, using the *rarefy_even_depth* function (rngseed = 1,121,983) in the phyloseq package [36] for R to compensate for uneven sequencing coverage prior to further statistical analyses. The relative read abundance (RRA) was visualised with bar plots for each dataset using the phyloseq and ggplot2 packages [37] in R, grouping the data according to sample origin (i.e. grass species) and sample type (i.e. false panicles and open panicles). Alpha diversity indices (Community richness: Observed richness [38] and Chao1 index [39]; Diversity: Shannon’s diversity index [40] and Simpson’s diversity index [41]) were estimated in the phyloseq package. Possible differences in alpha diversity indices between grass species and sample sites were assessed by a Kruskal–Wallis test [42]. The R package Rhea [43] was used to estimate beta diversity estimates using the Generalized Unifrac measure [44], which were visualized via NMDS plots and significant differences assessed via permutational multivariate ANOVA (PERMANOVA) analysis.

## Results

### Sequencing success and taxonomy

A total of 796 350 raw sequence reads were generated prior to filtering. Following denoising, chimera removal, and feature filtering, 692 626 reads (87%) were retained. The majority of the reads (98%) were observed in six samples, with the remaining samples showing low read recovery, with less than 5 000 reads (range = 97–4 225). Seven samples retained less than 50% of their initial input (Supplementary Table S1). The sample rarefaction curves indicated that only three samples were sufficiently sampled for the Arthropoda analysis (FCP.3, FPC.1 and FPC.2), and four samples were sufficiently sampled for the Fungal analysis (FCP.3, FTT.4, FPC.1 and FPC.2; Supplementary Figure S1).

Following the abundance filtering and curation via LULU, a total of 124 ASVs, represented by 692 005 reads, were retained for taxonomic identification. These ASVs were then further clustered to 50 OTUs. Fourteen OTUs had no taxonomic assignments, five OTUs were identified as Chordata (*Cirripectes vanderbilti, Homo sapiens,* unknown Phasianidae and *Gallus gallus*) and three OTUs identified as taxa belonging to the family Poaceae. These 22 OTUs were subsequently removed from the datasets. For the 28 retained OTUs, seven OTUs were assigned to Arthropoda (246 027 reads) and the remaining 21 to Fungi (Ascomycota, n = 14; Basidiomycota, n = 6; Oomycota, n = 1; 304 456 reads).

**Fig. 2:**
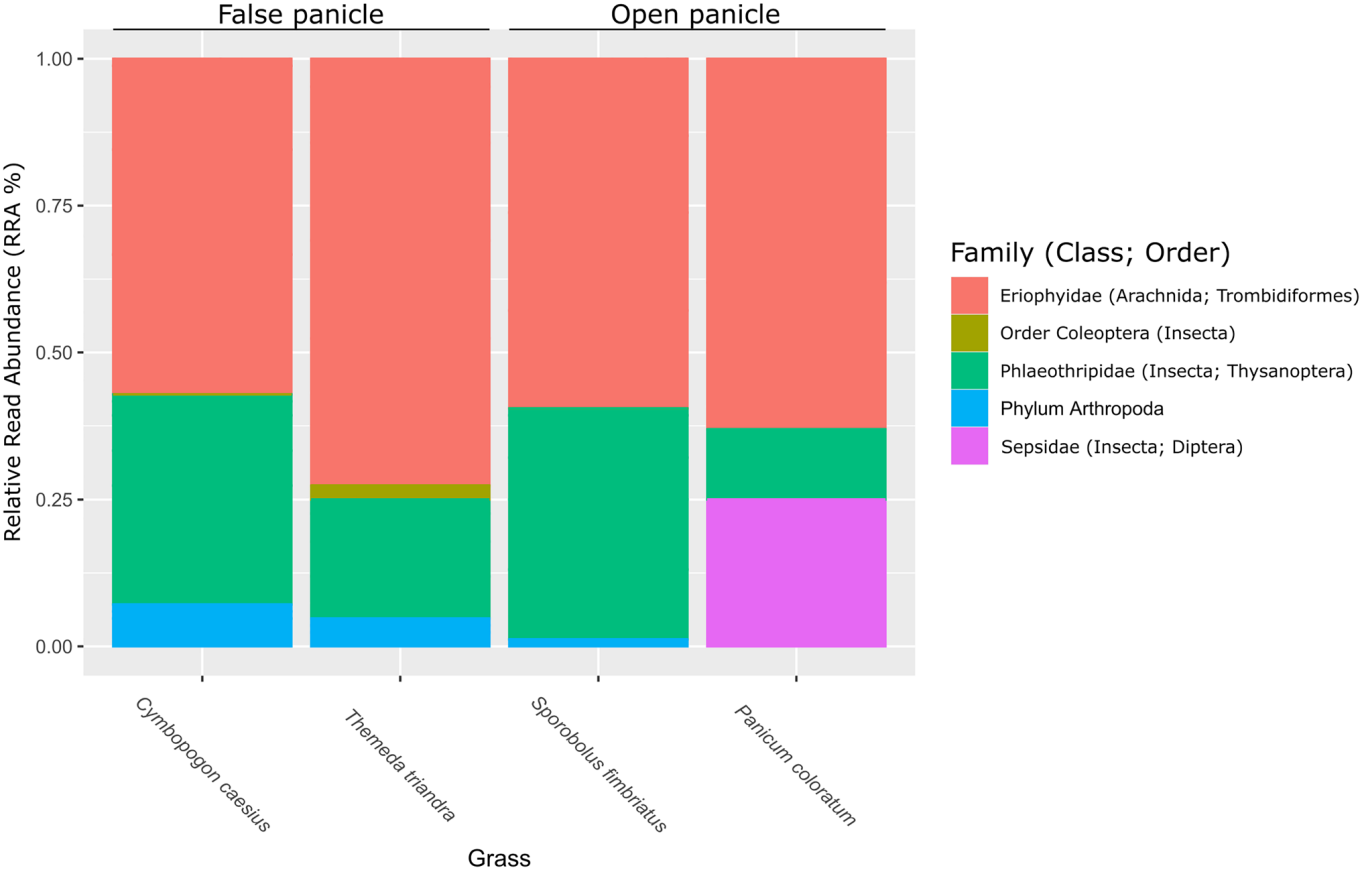
Relative read abundance (RRA) of the arthropod families and orders identified from the inflorescence surfaces of four grass species (*Cymbopogon caesius*, *Themeda triandra, Panicum coloratum, Sporobolus fimbriatus*). The dataset was rarefied to compensate for uneven sampling. The colour version of the image is available in the online version. (Color figure online)

The seven Arthropoda OTUs were assigned to four orders (Coleoptera, Diptera, Thysanoptera, Trombidiformes; Fig. 2). The lowest taxonomic placement for the Coleopteran OTU was order, with the Dipteran OTU assigned to *Sepsis flavimana*, the Thysanoptera OTUs were assigned to the genus *Haplothrips*, and the Trombidiformes OTUs formed part of the Eriophyidae Arachnid family. One OTU could only be identified as Arthropoda. Thysanoptera and Trombidiformes were observed in all sample groups (grass species). The most abundant group was represented by the Eriophyidae mites, which was observed in all samples (RRA = 57.1%—72.6%). In the normalized dataset, the Coleopteran and Dipteran OTUs were only observed in one sample each (Coleoptera—*T. triandra* RRA = 2.4%; Diptera—*P. coloratum* RRA = 25%).

**Fig. 3:**
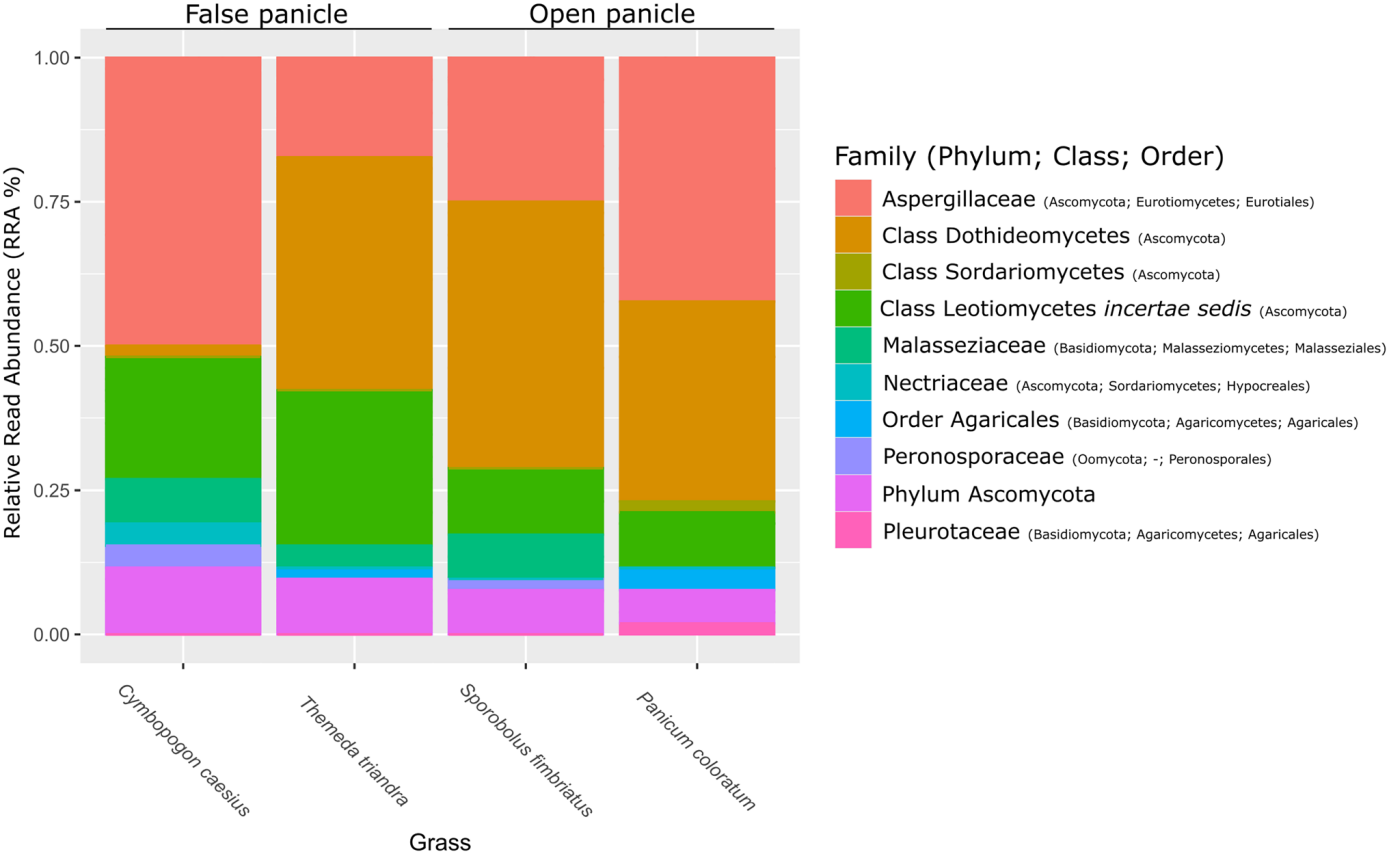
The relative read abundance (RRA) estimates for the fungal classes identified from the inflorescence of four grass species (*Cymbopogon caesius*, *Panicum coloratum, Sporobolus fimbriatus*, *Themeda triandra*). The dataset was rarefied to compensate for uneven sampling, with four OTUs removed. The colour version of the image is available in the online version. (Color figure online)

The Ascomycota OTUs (n = 14) were assigned to four classes (Dothideomycetes, Eurotiomycetes, Leotiomycetes and Sordariomycetes). The two Eurotiomycetes OTUs were assigned to the genus *Penicillium*, with the three Leotiomycetes assigned to the genus *Leohumicola* and two of these were further identified as *L. cerrucosa*. The three Dothideomycetes OTUs and one of the Sordariomycetes OTUs only assigned to class level, with the other Sordariomycetes OTU assigned to the family Nectriaceae. The most abundant classes were Dothideomycetes and Eurotiomycetes, which were observed in all four grass species groups. It was observed that Eurotiomycetes, and specifically *Penicillium*, had the highest RRA in the *C. caesius* samples, and an unknown taxon of Dothideomycetes were more dominant in the *T. triandra* and *S. fimbriatus* samples (Fig. 3; Supplementary Figure S2). Three classes of Basidiomycota were observed, with OTUs assigned to Agaricomycetes, Malasseziomycetes and Tremellomycetes. Two Agaricomycetes OTUs were identified to genus level as *Pleurotus* sp., with the Malasseziomycetes were assigned to the genus *Malassezia* sp. A single Oomycota OTU was assigned to the family Albuginaceae.

### Diversity

**Fig. 4:**
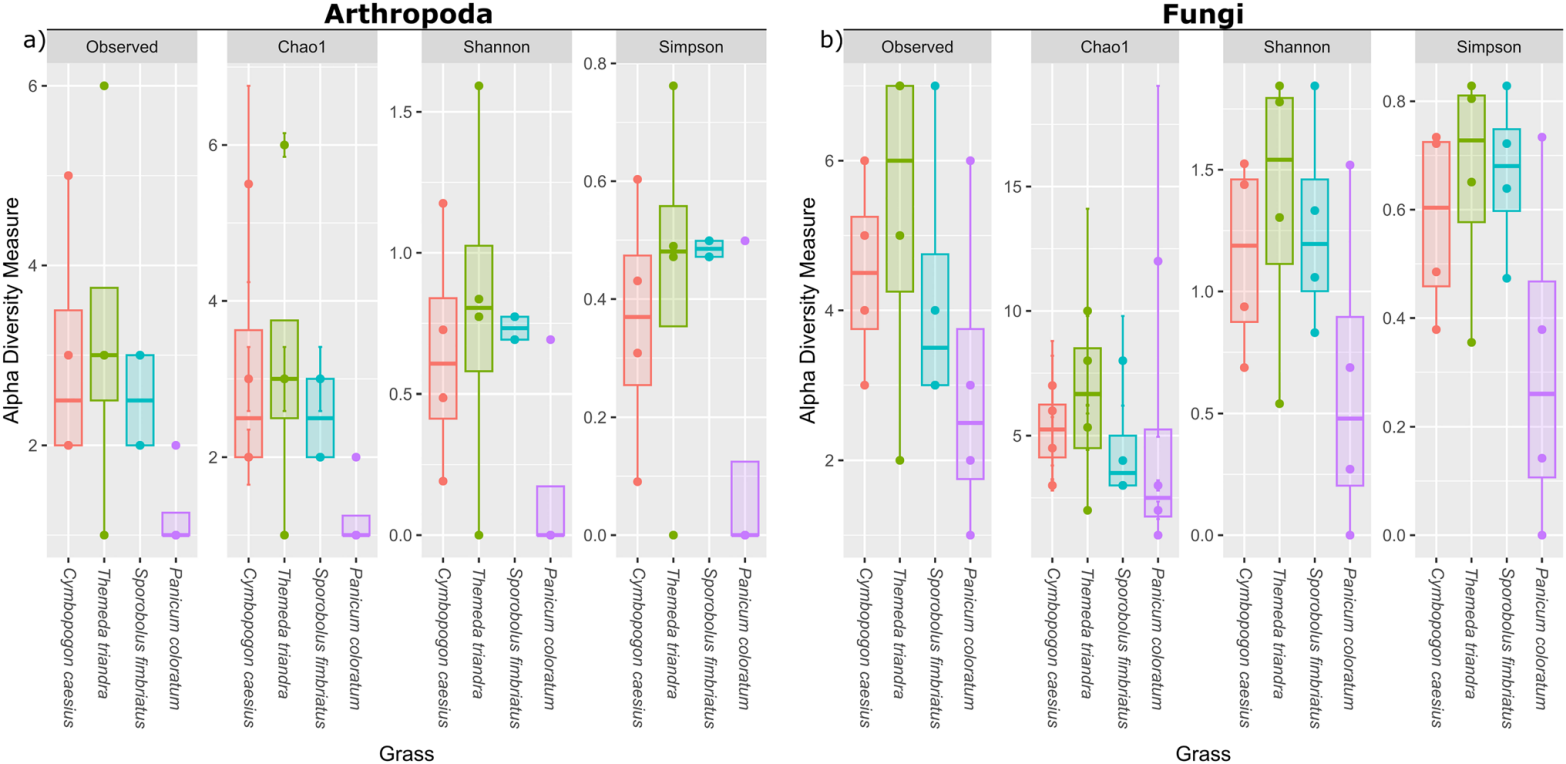
Alpha diversity indices (Observed Richness, Chao1, Shannon’s diversity index and Simpson’s diversity index) for the **a** Arthropoda and **b** Fungi datasets. The estimates were calculated from rarefied datasets. The groups represent the grass species from which the samples were taken (*Cymbopogon caesius*—red; *Themeda triandra*—green; *Sporobolus fimbriatus*—blue; *Panicum coloratum*—purple). The colour version of the image is available in the online version. (Color figure online)

The alpha diversities per grass species sampled can be observed in Fig. 4. Although no significant differences were seen between the grass species following the Kruskal–Wallis test, one can observe a general trend in the data. The alpha diversities for the *P. coloratum* samples were consistently lower in both datasets compared to the other grass species. The median values for the *C. caesius* and *T. triandra* samples were generally higher than for the open panicle grass species. The Generalized Unifrac measures for beta diversity showed no significant differences between grass species sampled (*p*-value > 0.05). The NMDS plot showed a clear overlap between all four grass species groups for both datasets (Supplementary Figure S3).

## Discussion

This study assessed the use of grass inflorescence for biodiversity monitoring within an urban grassland environment. The rarefaction curves suggest that additional sampling is required to capture a more significant portion of the biodiversity. The number of raw sequences obtained was also lower than expected. An explanation for this observation could be two-fold: the low amount of surface DNA on the grass inflorescence or the PCR biases of the chosen marker and PCR primers. The DNA concentrations could be improved by increasing the amount of extraction material used, by increasing the number of samples collected per site and performing DNA extraction replicates. The number of replicate samples needed will have to be assessed in a further investigation. The subsequent pooling of the replicate extracts could increase the amount of environmental DNA for PCR amplification. Increasing the number of replicate PCR reactions could further improve the detection of low-copy number DNA, and using shorter target loci (< 300bp) to better detect degraded DNA could additionally improve detection rates [13]. Gamonal Gomez et al. [13] further suggested using taxon-specific primers to improve targeted species detection and decrease the number of non-target taxa, such as the large number of fungal sequences observed in the current study. High abundances of fungal and bacterial taxa were reported by Lopes et al. [45] using the same COI primer set as in the current study. The occurrence of such high levels of microorganisms was deemed expected due to the nature of the sample material—faecal pellets. Ritter et al. [46] similarly reported that the majority of the observed COI sequence data generated from soil, plant and bulk insect samples were assigned to the fungal phyla Ascomycota and Basidiomycota. These authors targeted the same COI sequence region as in the current study but using modified primer sequences. Future studies should consider the broad range of taxa amplifiable from these primer sets and select a combination of primers and markers to improve the possible detections observed in line with the study objectives. Shorter Arthropoda specific primers, targeting COI [157 bp; [47] or 16S rRNA [157 bp; [48], would provide targeted results. Metagenomic shotgun sequencing of the eDNA extracts could be an alternative if funding is available, using state of the art sequencers such as the Illumina Novaseq or Oxford Nanopore PromethION systems. This metagenomic method can be performed without PCR amplification, thereby removing the PCR and primer bias concerns. The generated data would be based on the total DNA composition of the sample and not just a fraction like in a targeted approach and could provide composition data on the total ecosystem biodiversity ranging from viruses to bacteria to eukaryotes [49, 50]. A shortcoming of the current study design was identified after data generation and analysis were completed, which is the lack of environmental negative controls to account for the possible deposition of airborne eDNA on the plant surfaces. It should additionally be noted that DNA extraction blanks were not carried through to sequencing. The results reported here should, therefore, be considered with caution.

Despite the low sequence numbers and the small number of OTUs, several known grass-associated taxa were identified. Trombidiformes mites from the family Eriophyidae were dominant on all grass species. The members from Eriophyidae are known to occur on various species of grasses worldwide [51, 52]. These mites are obligate plant parasites, with several species causing damage to agricultural and ornamental plant species [53, 54]. Eriophyid mites have also been shown to serve as a valid biological control of invasive weeds, as seen in the case of the release of *Aceria malherbae* in South Africa to control the weed *Convolvulus arvensis* [55]. The second most abundant Arthropoda order was identified as Thysanoptera, exclusively represented by the genus *Haplothrips* (Phlaeothripidae), and was also observed on all grass species. The largest abundances were observed for the grasses *C. caesius* and *S. fimbriatus*. Members of the genus *Haplothrips* are known to live in the inflorescence of several plant taxa in South Africa, including grasses [56]. The diet of *Haplothrips* species include the eggs of spider mites (Tetranychidae, Trombidiformes) and eriophyid mites (Eriophyidae, Trombidiformes), as well as plants such as grasses [57, 58]. Thysanoptera is regularly reported in similar studies using plant inflorescence as eDNA source [12, 59–61]. An unidentified Coleopteran OTU was observed at low abundances for the assessed grass species. Members of the tribe Alticini are known phytophagous insects, with several species from the genera *Stegnaspea* and *Chaetocnema* known to be associated with grasses [62, 63]. Some members of the family Chrysomelidae have also been identified as possible biological control agents [64]. The Arthropoda taxa identified in the current study were also identified by Morrison et al. [65] while surveying for possible biocontrol candidates against Kenyan buffelgrass (*Cenchrus ciliaris*). Although RRA estimates have been suggested as an accurate method to view sample composition during metabarcoding studies, biases still exist [66]. The sole Dipteran OTU was identified as *Sepsis flavimana*. The distribution of this fly species is, however, restricted to the northern hemisphere [67]. This OTU is probably a member of the *Sepsis* genus, and closely related to *S. flavimana*. There are 43 *Sepsis* species known from the Afrotropical Region, with *S. stenocalyptrata* and *S. thorcica* previously observed in South Africa [68]. Sepsidae is a small fly family, showing morphological uniformity, mainly associated with animal dung [68; and references therein]. Some Sepsidae species have also been identified as possible pollinators [69]. The high abundances of Eriophyidae and Phlaeothripidae observed could be linked to skewed sampling. It is possible that invertebrate specimens were missed during the inflorescence inspection before DNA extraction due to the minute size of these arthropods. Thomsen and Sigsgaard [12] similarly suggested that the abundance of thrips observed in their study could be linked to the accidental inclusion of insect eggs, larvae or minute adult insects. Accidentally, including invertebrates in the extraction substrate would skew the amount of DNA available for PCR amplification, resulting in sequence read abundance values biased toward the included invertebrate taxa.

In the current study, a large number of COI sequences were identified as fungi. The phylum Ascomycota dominated the fungal dataset in the present study, and this supports previous reports of grass fungal diversity [70–72]. Reports on the fungal community composition for grass foliar tissues are globally highly diverse. For the current study, the fungal class Dothideomycetes was observed in all grass taxa*,* with low RRA in *C. caesius* samples, and Eurotiomycetes were more abundant in *C. caesius* and *P. coloratum*. Dothideomycete taxa were reported by Chen et al. [73] to have the greatest abundance on asymptomatic leaves of the South and North American native, *Sporobolus indicus*, similar to what was observed for *S. fimbriatus* in the current study. Dothideomycetes species such as the members of the genus *Cladosporium* is assumed to be a host-generalist and has been identified on leaf and stem tissues from several grass taxa worldwide [74–76]. *Penicillium sp.* (Eurotiomycetes) are well-known contaminants of foods such as grains and spices [77, 78], and the occurrence of this taxon on all grass species sampled here is unsurprising. High RRA values were observed for *Penicillium* in the *Cymbopogon caesius* samples included in the current study. This fungal genus has previously been reported as a highly abundant fungal taxon on another African *Cymbopogon* species, namely *C. schoenanthus*, from a study in Egypt [77]. The root endophytic genus *Leohumicola* (Ascomycota) was observed in higher abundances for the false panicle compared to the open panicle grasses. *Leohumicola* is regularly observed in grassland soils, and frequently associated with the grass rhizosphere [79, 80]. The Basidiomycete *Malassezia* is the sole genus of the class Malasseziomycetes, which is known to naturally occur on the skin surface of many animal species, including humans [81]. This could be either a sign of human mediated contamination during the field or laboratory work, or this could be as a results of carry over from fauna found in the area. It should be noted that these fungal observations are based on limited data generated from COI sequencing. Further investigations using fungi-specific genetic markers (e.g. ITS2) would provide more accurate fungal community data of these grass species. Including negative field control samples should also be implemented, as airborne fungal spores could also contribute to the DNA found on plant surfaces, thus increasing the chances of false positives.

The higher levels of arthropod and fungal alpha diversity indices observed for the *C. caesius* and *T. triandra* samples could be explained by the complex inflorescence structures of these grasses. The complex hairy inflorescence and large spikelets of *C. caesius* and *T. triandra* could potentially capture environmental DNA more effectively than the open panicle inflorescence and small spikelets of *S. fimbriatus* and *P. coloratum* [23]. Inflorescence size has previously been reported as a significant factor in the level of diversity detected using eDNA metabarcoding. Thomsen and Sigsgaard [12] similarly reported higher insect diversity levels from flowers with larger surface areas while assessing arthropod diversity using eDNA metabarcoding of various flower samples collected from dry grassland habitats in Denmark. More recently, while studying plant-pollinator interaction in western Australia, Newton et al. [61] also observed higher taxon detection rates for samples collected from plant species with large inflorescence. Grass endophytic fungi are known to have a broad host range, with some being generalists and others being host specifics based on host organ architecture [82]. The inflorescence of the false panicle grasses could also provide a suitable environment to support a higher fungal diversity, due to a potentially larger surface area for colonising the compound inflorescence structure with large spikelets, spathes and spatheoles. A further in-depth study of the fungal communities of these grass species could provide more details on the core and unique fungal taxa for these grasses.

Half of the taxonomic identifications of the Arthropoda OTUs were at higher taxonomic levels, with the lowest assignments to genus level for Diptera and Thysanoptera. The need for an in-depth reference database is crucial for accurate taxonomic identifications via eDNA metabarcoding [11]. The lack of lower taxonomic identifications for a large portion of the data is a strong indication of missing data in the reference database used, such reduced resolution is regularly reported in similar studies [61], and it is suggested that custom reference databases of local taxa should be constructed to improve genetic identifications [59]. A recent review did highlight the under representation of South African insect sequences in the BOLD database [33]. It is therefore important for South African researchers to address this shortcoming, which will improve the efficacy of eDNA metabarcoding during biomonitoring projects.

## Conclusion

The potential of using grass inflorescence as a source of environmental DNA to study metazoan and fungal biodiversity associated with different grass species has been highlighted in the current study. This study provides some new insights into the arthropod and fungal communities associated with four well-known grass species from central South Africa. However, the results should be considered with caution as extraction and PCR blanks were not carried through to sequencing. Negative field controls were also not included to account for the possibility of airborne eDNA contributing to the arthropod and fungal assemblages detected from these plant surfaces. Collecting additional replicates per grass species and increasing the DNA extraction effort could potentially further improve the HTS detection rates from grass inflorescence. It is additionally suggested that metabarcoding markers producing smaller amplicons with taxon-specific primers could provide improved HTS data during future studies of the biodiversity associated with grass species. If funding is available, one could also consider using metagenomic shotgun sequencing of eDNA to assess the total biodiversity of the samples. Such information could shine light on the occurrence of possible plant pathogens and parasites, as well as their associated predator species. Such data could also provide information on the presence of invasive species. Insect-mediated pollination does occur in some grass species, and a more in-depth study of arthropod diversity could identify important pollinator species. Further studies on grass-insect interactions and grass fungal diversity in South African grasslands can assist in future conservation management planning of these habitats. Increased effort should be placed on the improvement of local arthropod sequence representation on public reference databases such as BOLD and GenBank, to ensure accurate species identification in further South African studies.

## Supplementary Information

Below is the link to the electronic supplementary material.

Supplementary file1 (PDF 418 KB)

Supplementary file2 (XLSX 11 KB)

## Data Availability

All raw sequences are stored under BioProject PRJNA935099 (Assession numbers SAMN33297822-SAMN33297837) on the NCBI GenBank database.
