# Supplementary material for: Using grass inflorescence as source material for biomonitoring through environmental DNA metabarcoding
Source: Mol Biol Rep. 2024 Sep 16;51(1):987. doi: 10.1007/s11033-024-09885-9 (PMC11405429; doi:10.1007/s11033-024-09885-9)
Supplement: Supplementary file 1 — Supplementary file1 (PDF 418 KB) [file 11033_2024_9885_MOESM1_ESM.pdf]

Supplementary Figures: **Using grass inflorescence as source material for biomonitoring through environmental DNA metabarcoding**

W.G. Coetzer[1,2]

[1] Department of Zoology and Entomology, University of Fort Hare, Alice, Eastern Cape, South Africa

[2] Department of Genetics, University of the Free State, Bloemfontein, Free State, South Africa

ORCID: https://orcid.org/0000-0003-2189-5539

E-mail: coetzerwg@outlook.com

Postal address: Department of Zoology and Entomology, University of Fort Hare, Private Bag X1314, Alice, 5700

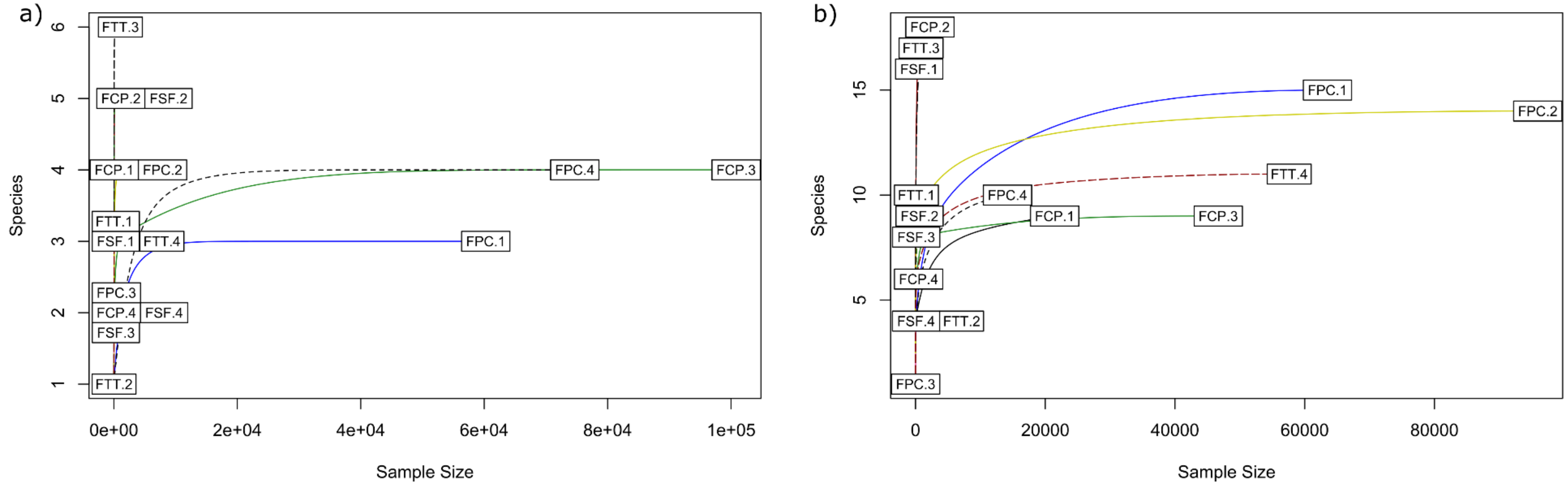

Supplementary Figure S1. The rarefaction curves for the high-throughput sequencing performed for the a) Arthropoda and b) Fungi COI datasets from the current study. The abbreviations are indicative of the four grass species sampled for environmental DNA during this study (FCC - *Cymbopogon caesius*; FTT - *Themeda triandra*; FPC - *Panicum coloratum*; FSF - *Sporobolus fimbriatus*).

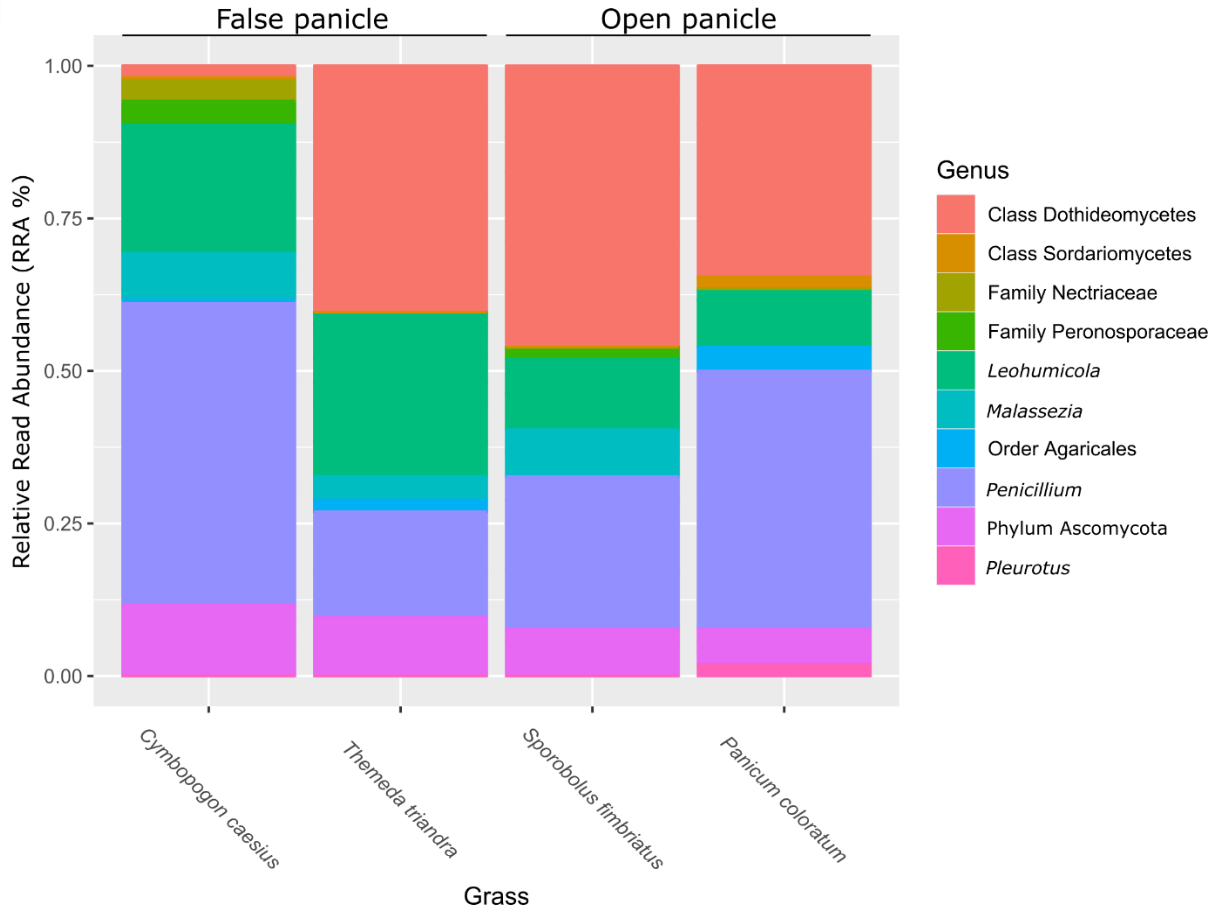

Supplementary Figure S2. The relative read abundance (RRA) plots per grass species indicating the proportions of possible genera in the dataset. Only three genera could be identified with certainty (*Cladosporium, Filobasidium, Penicillium*).

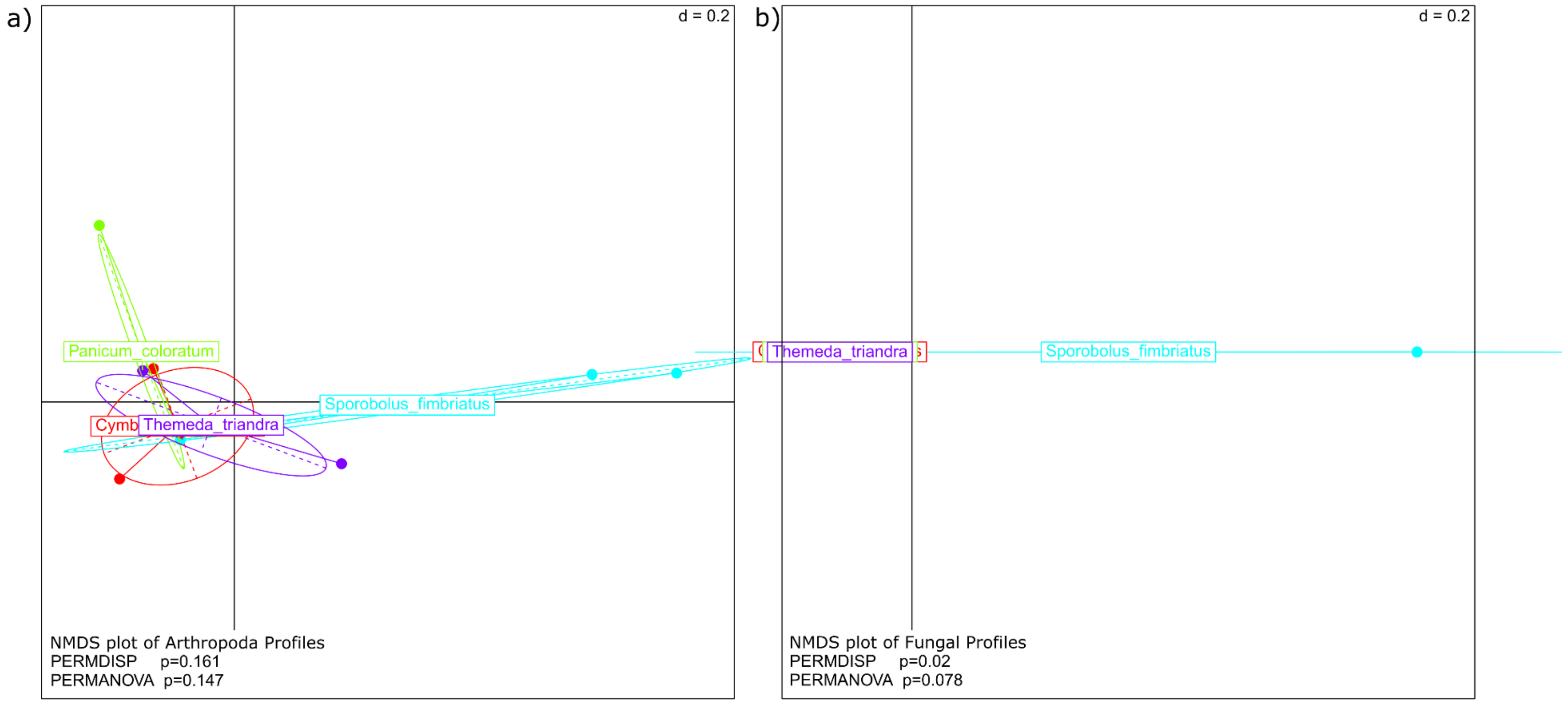

Supplementary Figure S3. Nonmetric multidimensional scaling (NMDS) plot for a) Arthropoda and b) Fungi datasets as estimated from the Generalized Unifrac measures calculated in the R (R Core Team, 2021) package Rhea (Chen et al., 2012).

References

Chen J, Bittinger K, Charlson ES, Hoffmann C, Lewis J, Wu GD, Collman RG, Bushman FD, Li H. 2012. Associating microbiome composition with environmental covariates using generalized UniFrac distances. *Bioinformatics* **28:** 2106-2113.

R Core Team. 2021. R: A language and environment for statistical computing. Vienna, Austria: R Foundation for Statistical Computing.
